# Refractive index gradient in the lens: reflections on form and function and on the lens paradox

**DOI:** 10.3389/fopht.2025.1709563

**Published:** 2025-11-17

**Authors:** Barbara K. Pierscionek

**Affiliations:** Faculty of Health, Medicine and Social Care, Medical Technology Research Centre, Anglia Ruskin University, Chelmsford, United Kingdom

**Keywords:** eye lens, refractive index, proteins, structure/function, lens paradox

## Abstract

**Introduction:**

The eye lens is a sophisticated optical element that provides the eye with both refractive power and transparency as well as the ability to change focus. The latter function diminishes with age as the lens becomes less able to change its shape. The changes with age in lens structure affect its function as a transparent refractive element but much remains misunderstood.

**Methods:**

The review considers the optical parameters of the lens, its gradient of refractive index, and how this may be formed and altered with growth and ageing. The review is structured around three axioms that relate to the creation of the refractive index, the explanation for the lens paradox, and the changes in the structural proteins and how these may be linked to opacification.

**Results/discussion:**

It is accepted that the structure/function relationship in the eye lens is explained by the distribution of its proteins forming a gradient of refractive index that provides a high level of image quality to the eye. Delving deeper into explanations for the gradient index creation, the lens paradox and the state of proteins *in situ* in lenses with cataract, gives reason for doubt. The axioms described indicate which areas require revisiting the literature, reconsideration of accepted thinking, and further experimental investigations.

## Introduction

The growth mode of the eye lens, which is an accrual of cell layers over existing tissue forming a lamellar structure with variations in protein concentration and distribution, creates a sophisticated optical structure with a gradient of refractive index (reviewed in 1). A linear relationship between protein concentration and refractive index provides a basic explanation of the structure/function link between the cytosolic proteins and the refractive index (reviewed in 1). Yet, this masks a deep complexity in the dynamic structure/function relationship that is still not understood. The nature of this relationship can be considered from a temporal perspective: how structure affects function during short-term changes with accommodation and with long-term changes that occur with age and that can lead to opacification.

Fundamental questions remain: i) what is the structural basis for the refractive index gradient; ii) how is it altered when the lens changes shape in the process of accommodation; and iii) how does the relationship between proteins and water alter with age and affect the optical properties?

The answers to these questions could benefit from novel methodologies that can image the microstructure without interference with its function. They also require a thorough re-examination of certain axioms. Some accepted notions about lens structure/function relationships have insufficient evidence or basis in fact and/or lack a convincing explanation.

## Axiom 1: that compression caused by growth creates the refractive index gradient

The notion of compression of existing tissue by new cell layers synthesised over older fibre cells, leading to compaction of cells in the lens nucleus, has been proposed (2) and accepted by many as an explanation for the refractive index gradient. This implies that compression causes the squeezing out of water and consequent increase in concentration of proteins in inner, older cells. Whilst appearing feasible, a mechanism for compression and the existence of any active force that may produce compression have never been postulated. The mere laying down of new cells on existing cells is not evidence of any such force and is insufficient to create a gradient of protein concentration that is the basis of the refractive index gradient. Indeed, in piscine lenses, findings show no evidence of compressed inner cell layers (3, 4).

It could be argued that compression is selective and only occurs in certain species. In the eye of an aquatic animal, the corneal power is effectively negligible given the very small difference between the refractive index of water and that of the cornea. The aquatic lens, therefore, needs to meet all of the demands of refractive power, unlike the terrestrial eye, in which the cornea contributes the greatest amount of refractive power to the eye. It is also notable that all piscine lenses examined to date are spherical or almost spherical (5–12). The gradient index is extremely important in such lenses because the spherical shape would lead to high levels of spherical aberration should the lens have a homogeneous refractive index. The gradient of refractive index in piscine lenses is steeper than in many terrestrial lenses (reviewed in 1). In addition, the magnitude of maximum refractive index in the centre of piscine lenses is amongst the highest of all species (5–12).

These differences between aquatic and terrestrial lenses—the greater refractive demand and the steeper refractive index gradient with the higher maximum refractive index in the former (reviewed in 1)—do not provide any insights into the formation of the gradient of refractive index nor any explanation for a selective mechanism of compression. Conversely, it provides an even stronger rebuttal to the concept of compression. If indeed compression created a gradient of refractive index, the steeper this gradient and the higher the magnitude of refractive index in the centre of the lens, the greater the compression should be. Yet, in piscine lenses with parabolic profiles and relatively steep gradients of refractive index, there is no evidence of compression (3, 4).

Given that a lamellar structure and the growth mode of tissue accrual on the surface have been found in all eye lenses thus far examined (reviewed in 1), there is no cause to believe that compression should occur in any species simply because of its growth mode or during early developmental stages. No compression was found in measurements of developing refractive index in embryonic chicken lenses (13). The assumption that there is compaction of lens fibres as the lens grows and ages suggests that the lens is compressible. Indeed, some studies have reported that lens volume may alter slightly with accommodation (14–16), which would support such a notion. Yet, these findings are not conclusive and are unsupported by results of other studies (17, 18). It is worth noting that a study of *in vitro* human lenses reported evidence of compaction of inner-layer fibres with age (19). These lenses had undergone fixation, which causes some dehydration and hence a loss of free water, which increases with age (20). More water will have been lost from older lenses. Furthermore, lenses were not in similar states of accommodation. If it is assumed that the post-mortem lens, released from any stretching forces imparted by the ciliary muscle, is in its most relaxed state, younger lenses will be in a state of accommodation, whilst older lenses, which have lost this functional capacity, will be unaccommodated. It is not possible therefore to make a comparison of fibre thicknesses if the accommodative states are not the same.

There are two other reasons why compression caused by cell accrual may be difficult to explain. Firstly, since cell layers are synthesised on the lens surface, if compaction occurred, this should be seen, at least initially, in the extreme periphery of the lens, resulting in an increase in refractive index at the lens edge and then a decrease with progression into the cortex. This has never been found and would be extremely detrimental to lens refractive function and to sight. Secondly, in lenses that accommodate, cell layers move. Such motion could therefore be expected to alter the amount of compression, if indeed cells could compress those in adjacent layers. This would alter local protein concentration and create kinks in the refractive index gradient. Yet, this does not happen. When the lens accommodates, the part of the lens where there is no gradient, which is approximately the nuclear section, changes in length; the gradient steepness in the cortex remains unchanged (21, 22).

An interesting observation was made some years ago about the potential link to the magnitude of refractive index and age (23). The comparison between two figures—one showing the equatorial radius plotted against age and the other showing radial distance from the lens centre plotted against (*n*_0_ − *n*(*r*))^2^ [where *n* is the maximum refractive index and *n*(*r*) is the refractive index at points along the lens equatorial radius]—showed remarkable similarity in shape (23). The first figure represented growth across a wide age range; the second showed differences in refractive index from the lens centre to points along the equatorial radius from a single older adult lens. Given that the lens grows by accrual of lens cells on the surface with no concomitant tissue loss, it is not unreasonable to assume that equatorial radius with age is akin to radial distance from the lens centre. In such a case, age and (*n*_0_ − *n*(*r*))^2^ would show a linear relationship: i.e., the difference in refractive index from the centre to the periphery would vary as the square root of age. If this is indeed the case, it is as yet unclear what this means. It could be indicative of a genetic programme within the growth mode of the lens such that changes in protein concentration as the lens continues to accrue new cell layers decrease with the square root of age in order to create the profile of refractive index to meet the optical demands of the eye. A caveat to the above assumption is that the human lens is not a sphere but rather that axial and radial symmetries and distances differ. The growth mode of the lens, which produces its lamellar structure, has been the basis of the assumption that the three-dimensional distribution of refractive index is as isoindicial contours (24). This has been applied in ray tracing in order to make measurements in the symmetric equatorial plane, transposable to the sagittal plane (24). From a basic perspective, this is reasonable as refractive index profiles in the equatorial and sagittal planes are similar: with a relatively flat central region and a steep change in refractive index in the cortex. From a more detailed analysis, power laws fitted to equatorial and sagittal refractive index profiles have shown some variation in the power law exponents (25). Whilst ranges are similar for lenses up to the sixth decade, greater scatter and higher exponent values were found for equatorial compared to sagittal fits applied to older lenses (25). The assumption of isoindicial contours in human lenses of all ages needs to be re-examined.

## Axiom 2: that the lens paradox can be explained by a decrease in central refractive index

Some years ago, classical thinking that the lens curvature must decrease with age was disproved by slit lamp studies that found the opposite (26, 27). This led to the apparent lens paradox, which described an apparent contradiction: that whilst the lens was growing and becoming more curved with age, the refractive power of the eye was not increasing (22, 27). A more curved lens should provide more refractive power, and given that the contribution of corneal refraction was not decreasing, there was no explanation for why the eye was not moving towards a more myopic state with age. The fact that the refractive index also contributes to refractive power was largely overlooked.

At that time, there had not been any means of measuring the refractive index in the intact lens. A decade later, Campbell and Hughes (28), using the mathematical treatise of Chu (29) for determining the refractive index of optical fibres using ray tracing, published the results showing the refractive index in the rat lens. The method was adapted to the human lens using additional mathematical methods to take into account asymmetries in curvature between the anterior and posterior surfaces of the lens (24). The results showed that the human lens only has a refractive index gradient in the outer, cortical part of the lens (23–25). The central nuclear section has an almost constant refractive index (23–25). This profile in the human lens, which can be fitted with a higher order polynomial, has been found in studies that have measured refractive index using ray tracing (24), fibre optic reflectometry (23), Pulfrich refractometry on a bisected lens (30), and X-ray Talbot interferometry (25). It is supported by studies on protein concentration (31) and water gradients (32, 33). It should be noted that these latter studies measured total water and hence can provide an accurate comparison to measurements of refractive index.

With age, the gradient steepness in the cortex changes (24, 34). These findings led Pierscionek to propose the first feasible explanation for the lens paradox: that the changes in the cortical refractive index gradient with age would lead to a small but significant decrease in the equivalent refractive index with age and that would be sufficient to offset the increase in curvature in order to retain the refractive power of the eye for distant vision (34). This was subsequently modelled and found to support the theory (35). Further support for this hypothesis was found in a study using Scheimpflug images from *in vivo* lenses (36) and in a more recent modelling study (37).

This hypothesis not only addressed the lens paradox with a feasible explanation but also took into account of what may happen when the lens accommodates (34). It has been shown that as the lens accommodates, only the nucleus widens along the optic axis with no change in cortical thickness (21, 22). Hence, there should be no alteration in the gradient of refractive index in the cortex, rendering a change in lens power during accommodation largely a result of lens shape change (34).

More recent studies have proposed that the lens paradox may be explained by a reduction in refractive index in the centre of the lens (38). This conclusion was reached based on measurements from *in vivo* eyes using MRI (38). Refractive index cannot be measured using MRI because MRI cannot directly measure total water; it measures the precessing of hydrogen nuclei and relaxation times as the tissues return to equilibrium (39). Free water has a relatively long relaxation time that can be detected; bound water does not and cannot be easily measured, if at all (39). Refractive index depends on the concentration of proteins and the total water in the lens. This includes water bound to proteins and water that is unbound or free. The former modulates protein conformation as well as dynamics and, hence, will have an impact on the refractive index and on the optical properties of the lens. To suggest a decrease in refractive index with age is to suggest that the lens is either losing protein or imbibing water as it ages. Neither of these processes can occur in a healthy lens and, indeed, would be detrimental for maintaining lens transparency. Free water, however, increases with age in the lens because water bound to protein is released (20). It is not surprising then that a study that has estimated refractive index from measurements that detect free water has reported an apparent decrease in refractive index with age. MRI is an excellent technique for investigating tissues in the natural state and can provide information about lens free water, but it is not a technique that will provide accurate measurements of lenticular refractive index. Donaldson and colleagues have attempted to infer total water from MRI measurements using a calibration against a tube of water at room temperature (40). Whilst indirect, this was an elegant and simple estimation that suggested that total water does not change with age in adult life (40), supporting a number of previous studies (41–43). Conversely, some investigations have reported a slight decrease in water content with age (20, 44). This would result in an increase in refractive index. These reported decreases were, however, at a very low level: approximately 2% between the second and ninth decades of life (20, 44).

Measurements of refractive index in post-mortem lenses using X-ray interferometry also suggested a slight decrease in maximum refractive index with age (25). It should be noted that this was not a value taken across the nuclear plateau but was the single highest value of refractive index. Furthermore, individual variations masked ageing trends with wide variations in lenses from below the fifth decade; no statistically significant age-related changes in refractive index were seen when the data were split into cohorts between ages of 20–60 and 60–90 years (25). There is another point that is often overlooked. The central part of the refractive index profile in a human lens is relatively flat (25). Any overall change in refractive index magnitude in this part of the lens would have very little impact on the refractive power and optical function. Changes in refractive index that would lead to localised variations would create light scatter, which, if sufficiently large, would manifest as opacification. A degree of light scatter can be tolerated by the visual system, and small changes in refractive index may have such a subtle effect on transparency that vision is not impaired (45). The continuum of light scatter renders it difficult to determine when a lens should be considered cataractous. The cataractogenic load hypothesis recognises that a number of gradual modifications to structural components in the lens may have protective effects during development and in the course of ageing before reaching a stage that causes disturbance to vision (46). It would be interesting to explore this hypothesis further and determine which structural modifications can be tolerated and at which stage protective changes become detrimental to sight.

## Axion 3: that increased insolubilisation of proteins is directly linked to cataract

The underlying cause of the loss of transparency, which is known clinically as cataract, is ascribed to protein aggregation. The explanation is that as proteins age, they denature and aggregate, producing localised foci of relatively high refractive index adjacent to water-filled lacunae of low refractive index. The relatively abrupt difference in magnitude of refractive index between the protein aggregates and water causes light to scatter and hence disrupts its traversal through the lens. From the perspective of physics, this is a feasible explanation, and opacities in the lens attest to some form of disturbance in the protein/water organisation. It is also well documented that more insoluble protein is extracted from older than from younger lenses and that the amount of insoluble protein extracted from cataractous lenses is greater than from normal lenses of the same age (44, 47). Moreover, proteins extracted from the inner layers of the lens contain higher proportions of insoluble protein than those extracted from the outer layers of the lens (47–49). A number of earlier studies on whole lenses have shown that insoluble protein content increases with age (50–54). It is therefore reasonable to infer that insoluble proteins that have been extracted from the lens are linked to or are indeed a manifestation of the process of protein aggregation in the intact lens. However, very high levels of insoluble protein were found in the inner layers of a transparent piscine lens (reviewed in 1). The increase in insoluble protein extracted from human lenses with age does not equate to the rate of decrease in transparency; relatively high levels of insoluble protein have been extracted from older lenses, which do not have any manifest opacification (47, 55). As proteins in the lens age, they undergo conformational changes, which may render them more vulnerable to insolubilisation on extraction from the lens. This may not necessarily be a manifestation of aggregation and opacification *in situ*.

Changes in protein conformation may cause subtle local alterations in the organisation between proteins and water that are not sufficiently disruptive to vision for the lens to be deemed cataractous. Yet, refractive changes to the eye can be evident. Nuclear cataract has been linked to a myopic shift (56–58). These myopic shifts have been observed before nuclear cataract is apparent (56, 57, 59–61). Increased nuclear density found in lenses with nuclear opacification indicates an increase in refractive index of the nucleus, which would explain the additional refractive power. How localised or generalised this increase is, is not clear. It is worth noting that the opacification in the nucleus is termed nuclear sclerosis (56) because there is a generalised hardening of the nucleus. It is well known that nuclear opacification also involves an overall colouration of the nucleus caused by absorption of shorter wavelengths of visible light. Attenuation of light by absorption cannot be explained by a localised high refractive index aggregates surrounded by lacunae. Whilst hardening and colouration are found in nuclear opacification and there is some broad correlation, the degrees of these features are not directly linked (62).

Proteins alter their natural conformation, and thereby their functions, in response to a wide range of stresses, both physical and chemical. The discovery of protein refolding after denaturation (for which Christian Anfinsen received the Nobel prize in chemistry in 1972) signified the importance of amino acid sequence on protein higher-order structures (reviewed by 63). It has prompted many subsequent investigations on causal factors that alter protein state. Thus far, observing such changes at the single protein level has been very difficult to achieve. Liquid-based atomic force microscopy has provided advanced imaging modalities that allow visualisation of conformational changes to single proteins in response to chemical stress (63). This does not address changes that occur within a tissue over short durations, such as what may occur when the lens accommodates, nor those that occur over many years such as those that lead to proteins becoming insoluble on extraction from the lens.

## Conclusion

The transitions that proteins undergo and the way that they alter their form when extracted from an intact lens will offer fundamental insights into what is occurring *in vivo*. How to detect such changes should be a focus of future research. It will demand increasingly greater advancements in imaging methods that do not interfere with or alter the protein conformation. This can be aided by computational modelling and predictions of protein state, which can be compared to experimental observations. The latter is an indirect method, as is any modelling approach, and depends for accuracy and relevance on reliable data used to construct the models.

Any conformational change in proteins is very likely to alter the protein/water relationship and be linked to the alterations in water state, i.e., more free and less bound water as the lens ages. This indicates that a certain amount of change in the balance of bound to free water is tolerated optically and does not lead to opacification. Future investigations should consider whether the change in water state is pathological, eventually leading to opacification or a natural process of ageing. If this is indeed linked to the process of cataract formation, just how much transition of bound to free water can be borne by the lens before opacification starts to occur and whether this can be controlled would provide important insights for cataract prevention and/or retardation.

Further research should also consider a spectrum of water states: between bound and free states, there will be water that interacts in different ways with protein—water that is partly bound and may remain so or may in time be free. It would be interesting to measure how such a diversity in water states may modulate magnetisation of the protons and the relaxation time seen in MRI measurements and whether this could be linked to vulnerability to insolubilisation and/or predict the development of opacification.

The relationship between the proteins and the refractive index gradient that they create by the mode and rate of growth of the lens requires further exploration. Is the refractive index gradient a manifestation of a genetic programme, an effect of growth, or both? If growth mode is indeed causal in the gradient index formation, what is the mechanism that creates such a gradient, and does this vary in different species? If it is genetic, does it vary with individuals, and how may this impact the refractive state of the eye? The importance of understanding this structure/function link cannot be underestimated. It could lead to a more sophisticated appreciation of the opto-biomechanics of the lens, what may expediate or retard lens ageing, and whether there is any potential control on the rate of accommodative loss or development of opacification. Finally, the renewed interest in the so-called ‘lens paradox’ would benefit greatly from a perusal of the literature and a better understanding of basic optics. Slight changes in refractive index in the nuclear region of the lens, if indeed they exist, could not offset the increased curvature of the lens with age and prevent an increase in refractive power of the eye because the nuclear region is one of relatively constant refractive index. The greatest contribution to refractive power from the material properties of the lens comes from the cortex, where there is a gradient of refractive index, and the changes in the slope of this gradient with age are sufficient to explain the ‘lens paradox’. Further research is needed into what may cause changes in the cortical refractive index with age and whether this varies depending on refractive error, ethnicity, or other genetic or environmental factors as well as how the underlying protein conformational changes alter the optical properties in various types of cataract.
